# An Unusual and Fatal Cause of Miliary Nodules on Chest Radiography

**DOI:** 10.3390/jcm7070164

**Published:** 2018-06-29

**Authors:** Anmol Cheema, Saira Chaughtai, Usman Mazahir, Manimala Roy, Mohammad A. Hossain

**Affiliations:** 1 Department of Medicine, Jersey Shore University Medical Center, Hackensack Meridian Health, 1945 State Route 33, Neptune, NJ 07753, USA; anmol.cheema@hackensackmeridian.org (A.C.); usman.mazahir@hackensackmeridian.org (U.M.); mohammad.hossain@hackensackmeridian.org (M.A.H.); 2 Department of Pathology, Jersey Shore University Medical Center, Hackensack Meridian Health, 1945 State Route 33, Neptune, NJ 07753, USA; manimala.roy@hackensackmeridian.org

**Keywords:** foreign body granulomatosis, talc, microcrystalline cellulose, intravenous drug use

## Abstract

Foreign body granulomatosis has many etiologies, including the injection of oral medications intravenously. The insoluble filler materials that are used in the medications can lodge in pulmonary arterioles and capillaries, which can trigger foreign body giant cell reaction, chronic inflammation, thrombosis, and fibrosis, resulting in pulmonary hypertension, progressive shortness of breath, and, potentially, fatal conditions. On imaging, this may present with multiple miliary mottling’s/nodules. The use of a bronchoscopy with biopsy can be an excellent way to establish a diagnosis in appropriate clinical settings. Here, we present a case of a 37-year-old old male found to have multiple miliary densities on imaging due to intravenous use of oral medication.

## 1. Introduction

Pulmonary foreign body granulomatosis can be caused by intravenous injection of grounded oral medications [1]. Oral medications are used to increase their action and potency. Typical medications abused in this manner include methylphenidate, opiates, antihistamines, and meperidine [2,3]. Oral medications typically contain insoluble agents that are meant to help with digestion. These agents, such as talc and microcrystalline cellulose, if found in the wrong tissues can cause an immune reaction. This clinical scenario is not common and there is no well-established treatment other than cessation of the offending agent. We report a case of a patient who presented with dyspnea and miliary densities in his lungs on computer tomography.

## 2. Case Report

**Figure 1:**
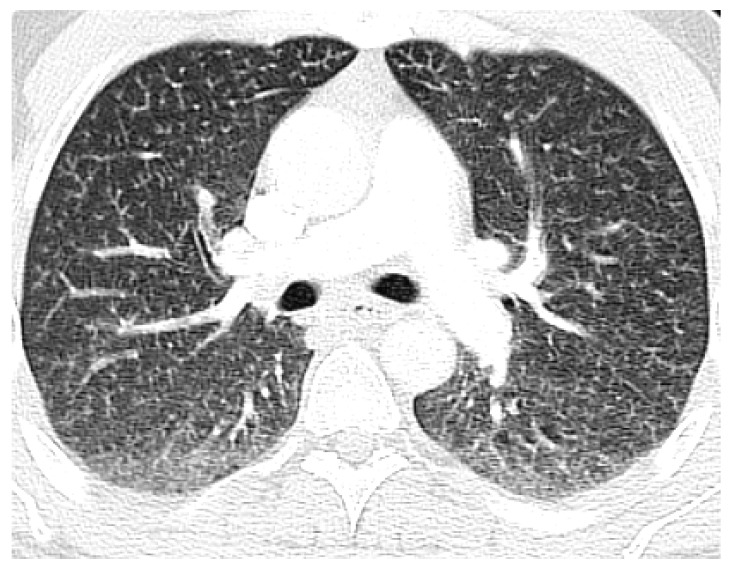
Axial computer tomography angiography (CTA) at the level pulmonary arteries showing extensive military densities in the bilateral lung fields with no evidence of embolism.

**Figure 2:**
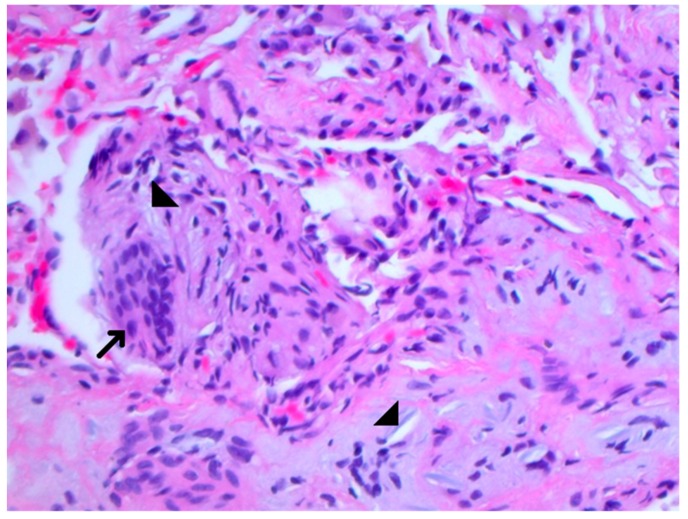
The transbronchial lung biopsy showing perivascular multinucleated foreign body giant cells (arrow) containing refractory material consistent with microcrystalline cellulose material (arrowheads). Formalin fixed paraffin embedded sections of a transbronchial lung biopsy are stained with Hematoxylin and eosin ×400 (H&E ×400).

A 37-year old homeless male, with a past medical history of peripheral vascular disease, type 1 diabetes mellitus, hypertension, and depression, presented to the emergency department with intermittent chest pain and progressive shortness of breath for a few weeks. The patient denied intravenous drug use, although admitted to the use of recreational marijuana. The patient’s vitals on admission were stable except for low saturation on a pulse oximetry of 86% on room air. On physical examination, the patient was tachypnic and had fine crackles in the bilateral lung fields on auscultation. Laboratory results on admission showed a normal complete blood count and basic metabolic panel, but a urine drug screen test was positive for opioids. Because of persistent hypoxia, a D-Dimer was checked and came back highly elevated. The patient underwent a computed tomography angiography (CTA) of the chest to rule out pulmonary embolism. The CTA was negative for pulmonary embolism, however, it displayed extensive miliary densities throughout the bilateral lung fields (Figure 1). The patient was admitted to the floor with a differential diagnosis of military tuberculosis versus fungal infection. Human immunodeficiency virus (HIV), fungal, and Quantiferron testing were negative. The cardiac work up and autoimmune serology were also unremarkable. The patient was then started on intravenous steroids and inhaled albuterol, although no improvement was seen. The patient remained hypoxemic despite therapy, and, therefore, underwent a bronchoscopy with a lung biopsy to find out the etiology of the disease process. The lung biopsy showed alveolated lung tissue with a miliary pattern of perivascular foreign body histiocytes containing refractory material suggestive of microcrystalline cellulose material (Figure 2). There was no evidence of malignancy and there were no fungal or acid fast bacilli organisms identified on special stains. The histological features suggested intravenous injection of foreign material and upon further questioning the patient admitted to injecting oral opiates. The patient was started on intravenous steroids, although his clinical condition continued to decline. The patient developed hypercapnic respiratory failure, which required intubation, and eventually suffered from a cardiopulmonary arrest and passed away.

## 3. Discussion

Oral medications contain insoluble particulate filler materials that bind and protect the drug during production, as well as shape and lubricate the tablet for swallowing [1,2,3]. Excipients include talc (magnesium silicate), microcrystalline cellulose, and starch [2,4,5,6,7]. Chronic intravenous drug abusers have been known to inject crushed pills that are intended for oral administration, as with our patient; he admitted to crushing pills, then mixing this with water and heating the mixture, and, finally, injecting himself [8,9]. The insoluble particles can lodge in pulmonary arterioles and capillaries, which can trigger chronic inflammation, thrombosis, and fibrosis. This can result in pulmonary hypertension and progressive shortness of breath, which is, potentially, fatal [10,11,12]. In time, microcrystalline cellulose and talc particles will form foreign bodies, which can provoke a histiocytic and foreign body giant cell reaction [9]. Although, compared with talc, the microcrystalline cellulose particles are larger and are less likely to pass through the lungs to other organs [13]. In our patient, there was extensive lung involvement with nodular densities, but there was no evidence of other organ involvement.

Our literature review demonstrated that patients who inject oral medications may be asymptomatic or have only nonspecific symptoms, with the most common complaint seen being shortness of breath [14]. Patients can abruptly develop pulmonary arterial hypertension, cor pulmonale, or even sudden death [2], which was observed in our patient. In these groups of patients, a pulmonary embolism should be first excluded as was done in our case with a D-Dimer and a CTA. The patients may also complain about experiencing recurrent episodes of respiratory decompensation and/or fever, which coincide with rounds of injection [15,16]. Most IV drug users deny drug abuse, as in our case, although some may have needle tracts on physical examination or a history of IV drug use [1,17]. The examining physician requires a high index of clinical suspicion for making the diagnosis in the early stages.

Computed tomography (CT) scan in microcrystalline cellulose granulomatosis shows nodules that are diffuse and small, involving primarily the mid zones. When the disease progresses, the small nodules will coalesce into large nodules [18,19]. In talc granulomatosis, the CT scan also shows mid-zone nodular interstitial disease, although there is also hilar and mediastinal adenopathy present [18,19]. Typically, a high-resolution CT is superior to a conventional radiography, and is able to show the nodules and their distribution better [18]. The differential diagnosis based on radiographic findings includes talcosis, miliary tuberculosis, fungal disease, and miliary metastases [18]. There are other diseases that can present with micronodules, such as sarcoidosis, extrinsic allergic alveolitis, and silicosis, although these are usually not in the top differentials [18]. When the clinical and radiologic findings are highly suggestive of foreign body granulomatosis further diagnostic evaluation is typically unnecessary, as long as the clinical course is stable. Although, when the diagnosis is unclear or the patient presents with acute symptoms, both of which were seen in our case, a bronchoscopy with a biopsy should be obtained to confirm the diagnosis [20,21].

There are no established guidelines for the treatment of foreign body granulomatosis, and the approach to management typically depends on the stages and pattern of disease and the severity of symptoms. The most important first step is the cessation of smoking and IV drug abuse. For patients that are asymptomatic, periodic reassessment for potential disease progression is recommended as the disease can progress to pulmonary fibrosis despite cessation of IV drug use [19]. For patients that have a chronic progressive disease with respiratory impairment, there is no established treatment. For patients that have acute onset of dyspnea, as was seen in our patient, improvement in symptoms and gas transfer may occur over days to weeks with supportive care alone, presumably due to the resolution of thrombi and clearance of injected material from the pulmonary circulation [22]. This is different then what was seen in our patient who rapidly declined even with supportive care and IV steroid use. Suggestions have been made to use systemic or inhaled glucocorticoids, or immunomodulating agents, to suppress granulomatous inflammation, although, data to support this is sparse [22,23,24]. If a patient develops pulmonary hypertension as a result of the disease, supportive therapy with oxygen and cautious diuretic use can be initiated [22]. Lung transplants for these patients has been performed in rare examples where alternative treatments failed [8]. The prognosis of this foreign body granulomatosis is poor due to complications from progressive interstitial lung disease, which can happen even years after cessation, pulmonary hypertension, and angiothrombosis [8].

## 4. Conclusions

Intravenous injection of oral medication causing foreign body granulomatosis of the lung is a complication that can be overlooked, especially in a patient with no history of IV drug use. The use of a bronchoscopy with a biopsy can be an excellent way to help in the diagnosis of this disease. The treatment both in the acute and chronic phase is not well established, and the main treatment is supporting and modulating the immune system. As is seen in our case, in the acute setting, the use of supportive treatment and steroids is typically recommended. The diagnosis of this unusual disease can be challenging. This differential may not be considered without a reliable history and a high index of clinical suspicion, especially in the appropriate setting by the treating physician.
